# Association between Sleep, Alzheimer’s, and Parkinson’s Disease

**DOI:** 10.3390/biology10111127

**Published:** 2021-11-03

**Authors:** Sumire Matsumoto, Tomomi Tsunematsu

**Affiliations:** 1 Advanced Interdisciplinary Research Division, Frontier Research Institute for Interdisciplinary Sciences, Tohoku University, Sendai 980-8578, Japan; sumire.matsumoto.e6@tohoku.ac.jp; 2 Super-Network Brain Physiology, Graduate School of Life Sciences, Tohoku University, Sendai 980-8577, Japan; 3 Precursory Research for Embryonic Science and Technology, Japan Science and Technology Agency, Kawaguchi 332-0012, Japan

**Keywords:** Alzheimer’s disease, Parkinson’s disease, β-amyloid, tau neurofibrillary tangles, α-synuclein, sleep, REM behavior disorder, orexin, EEG

## Abstract

**Simple Summary:**

Protein misfolding and aggregation in the brain are associated with various neurodegenerative diseases, including Alzheimer’s disease (AD) and Parkinson’s disease (PD). Sleep impairments are frequently observed in AD and PD patients not only as accompanying symptoms, but also as a prodrome that precedes the onset of these diseases. This suggests the potential of sleep impairments as a biomarker for the early diagnosis of, or as a target of, the treatment of these neurodegenerative diseases.

**Abstract:**

The majority of neurodegenerative diseases are pathologically associated with protein misfolding and aggregation. Alzheimer’s disease (AD) is a type of dementia that slowly affects memory and cognitive function, and is characterized by the aggregation of the β-amyloid protein and tau neurofibrillary tangles in the brain. Parkinson’s disease (PD) is a movement disorder typically resulting in rigidity and tremor, which is pathologically linked to the aggregation of α-synuclein, particularly in dopaminergic neurons in the midbrain. Sleep disorders commonly occur in AD and PD patients, and it can precede the onset of these diseases. For example, cognitively normal older individuals who have highly fragmented sleep had a 1.5-fold increased risk of subsequently developing AD. This suggests that sleep abnormalities may be a potential biomarker of these diseases. In this review, we describe the alterations of sleep in AD and PD, and discuss their potential in the early diagnosis of these diseases. We further discuss whether sleep disturbance could be a target for the treatment of these diseases.

## 1. Introduction

Neurodegenerative diseases are characterized by the progressive dysfunction and loss of neurons in the patients’ brains. These diseases are often associated with the misfolding and aggregation of aberrant proteins. Alzheimer’s disease (AD) and Parkinson’s disease (PD) are two major neurodegenerative diseases. The number of people affected by these diseases is currently increasing throughout the world. Approximately 50 million people worldwide have AD, and the number is expected to increase in the coming years. The total cost of care for AD patients is estimated to become more than $1 trillion by 2050 in the United States [1]. Approximately 1% to 2% of people older than 60 years, and 4% of those older than 80 years have PD [2]. These diseases remain incurable to date, and delaying their progression also remains difficult.

Mounting evidence has shown that sleep disturbances accompanying neurodegenerative disorders are common. Moreover, sleep has recently been gaining attention as a prodromal symptom and as a risk factor that exacerbates these neurodegenerative diseases. In this review, we describe the alterations in sleep observed in patients with AD and PD, and discuss whether sleep abnormalities can be used as a biomarker of these neurodegenerative diseases for early disease diagnosis, and as a possible treatment target.

## 2. AD and Abnormal Proteins

AD accounts for 60% to 70% of all cases of dementia, and shows the gradual impairment of the memory and cognitive function of patients. Pathologically, the accumulation of toxic forms of β-amyloid (Aβ) and tau neurofibrillary tangles in the brain are considered to be the main cause of AD.

Aβ is a proteolytically cleaved product of amyloid precursor protein (APP), and several variants with different carboxy termini have been identified. Aβ_1-42_ and Aβ_1-43_ are considered to have higher aggregation tendencies than Aβ_1-40_ [3,4]. Aβ is produced continuously even under healthy conditions, and increased Aβ deposition can be observed in some cognitively normal older individuals [5,6]. Several pathways for Aβ clearance exist, as follows: (1) enzyme degradation, (2) transcytosis, (3) intramural periarterial drainage, and (4) glymphatic system of the brain. Aβ aggregation appears to occur as a result of an imbalance between its production and clearance, predominantly owing to reduced Aβ discharge [7], although the precise reason for the absence of any pathogenic effects of Aβ burden in normal aging brains remains unclear. A previous report suggested that the abundance of the N-terminal truncated species of soluble Aβ in the aggregates correlate with its toxicity [6].

Tau is a microtubule (MIT)-associated protein that mainly exists in neuronal axons [8], which maintains MIT structure, and synaptic structure and function [9,10,11]. Under pathological conditions, the tau protein is hyperphosphorylated, and dissociates from MITs and starts aggregating and forming neurofibrillary tangles [12,13].

One hypothesis that has been widely considered as the basis of AD pathology is the amyloid cascade hypothesis [14]. This hypothesis proposes that the accumulation of Aβ is the initial event in AD pathogenesis, and is followed by tau neurofibrillary tangle accumulation [15], mitochondrial damage [16], and eventual neuronal death. Identification of mutations in the APP gene that cause increased Aβ deposition in familial AD support this hypothesis [17]. In addition, mutations in the presenilin (PS) genes *PS1* and *PS2* have also been identified as causative genes that enhance APP processing to generate toxic forms of Aβ [18].

Another model to explain the pathogenesis of AD is that soluble Aβ oligomers disrupt the glutamatergic synaptic function of neurons [19,20]. It has been reported that Aβ regulates the surface expression of the N-methyl-D-aspartate (NMDA)-type glutamate receptor through the α-7 nicotinic receptor, protein phosphatase 2B (PP2B), and striatal-enriched tyrosine phosphatase (STEP) [21]. Aβ activates STEP, which dephosphorylates Tyr1472 of the NMDA receptor subunit protein NR2B which results in endocytosis of the NMDA receptor [21,22,23]. Aβ can also induce the endocytosis of α-amino-3-hydroxy-5-methyl-4-isoxazolepropionic acid (AMPA)—type glutamate receptors [24,25].

The tau hyperphosphorylation hypothesis has also been proposed as a central pathogenesis of AD [26,27]. Tau deposition observed earlier than the aggregation of Aβ supports this hypothesis [28]. The phosphorylated tau proteins weaken the structure of the MITs, which eventually leads to necrosis or apoptosis. Hyperphosphorylated tau and tangles are also known to disrupt axonal transport [29,30].

While it is evident that accumulation of Aβ and tau tangles are pathological features of AD, all these hypotheses of AD development mechanism are not conclusive and the precise pathogenetic processes of AD development are yet unclear.

## 3. Aβ and Sleep Impairments in AD

In addition to memory and cognitive impairments, sleep disturbance commonly occurs in AD patients [31]. The clinical symptoms of AD dementia manifest progressively through subjective cognitive impairment (SCI), mild cognitive impairment (MCI), and mild to severe AD dementia [32]. More than 65% of AD and MCI patients have been reported to have at least one clinical sleep disorder [33].

**Figure 1:**
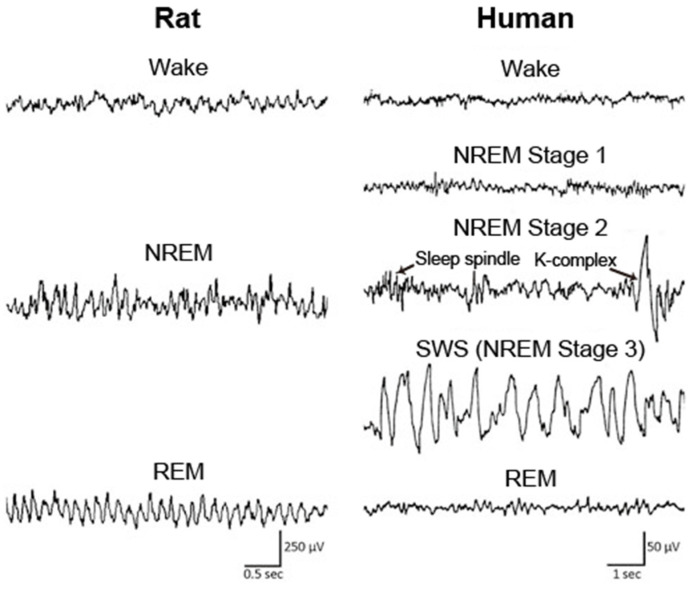
EEG across wake and sleep in human and rat. In rat, the sleep stage is classified into wake, NREM sleep, and REM sleep. In human, sleep is typically classified into NREM stage 1, NREM stage 2, NREM stage 3, and REM sleep according to the characteristics of EEG waveform, including sleep spindle and K-complex in NREM stage 2. NREM: non-rapid eye movement; REM: rapid eye movement; SWS: slow wave sleep. Figure adopted from [34] with permission.

Sleep is classified into the following four stages in humans: non-rapid eye movement (NREM) stage 1, NREM stage 2, slow wave sleep (SWS), and rapid eye movement (REM) sleep, according to the characteristic brain and muscle activities in each stage (Figure 1). In animals, including mice and rats, sleep is typically classified into the following two stages: NREM and REM sleep (Figure 1). During NREM sleep, cortical electroencephalogram (EEG) shows large amplitudes and slow oscillating waveforms in the delta (0.5–4 Hz) range, whereas low amplitudes and fast waves are predominantly observed during wakefulness and REM sleep. Muscle tone becomes extremely low during REM sleep, which is called REM atonia.

In healthy older individuals, well characterized sleep alterations are observed; i.e., a higher number of nocturnal awakenings and fragmentation of sleep, sleep instability, shorter daily sleep duration resulting from equal decreases in NREM sleep and REM sleep, increases in the proportions of NREM stages 1 and 2 (light sleep), less or shorter NREM/REM cycles, and decreased slow wave activity (SWA) in the spectrograms of EEG during NREM sleep [35,36,37,38]. In AD patients, sleep is altered to an extent that is greater than that observed in normal aging. The decrease in the percentage of SWS, REM sleep duration, and SWA in SWS EEG is significantly accelerated in AD patients compared with age-matched controls [31]. The density of K-complex and sleep spindle, specific features of NREM stage 2 (Figure 1), are poorly formed in AD patients [39]. AD patients also have a high prevalence of daytime sleeping [40]. In addition, AD is associated with a delay in the circadian phase, which is opposite to the typical advance in circadian phase that occurs upon normal aging [41]. These sleep disturbances in AD patients have substantial impacts on both the patients and their caregivers [42].

Recently, there has been increasing interest in the sleep impairments associated with AD, which are an important component of AD pathophysiology [43,44,45]. Additionally, accumulating evidence has shown that sleep alteration emerges before the clinical state of AD and insufficient sleep could be a risk factor of AD. Thus, it has been proposed that the association between sleep impairments and AD pathology is bidirectional [43,45].

**Figure 2:**
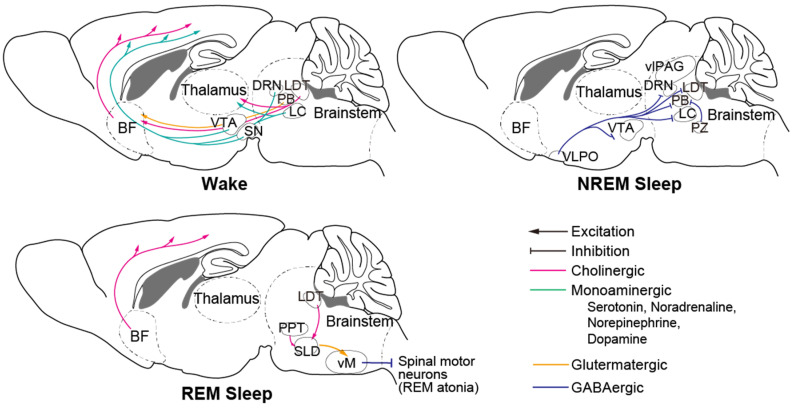
Principal neural circuit across wake and sleep. Monoaminergic neurons of wake-promoting nuclei in the brain stem innervate to thalamus and entire forebrain and activate during wake. During NREM sleep, NREM-active regions including PZ and VLPO inhibit those wake-promoting neurons. During REM sleep, SLD neurons activate vM neurons and inhibit spinal motor neurons that cause REM atonia. BF: basal forebrain; VTA: ventral tegmental area; SN: substantia nigra; DRN: dorsal raphe nucleus; LDT: laterodorsal tegmentum; PB: parabrachial nucleus; LC: locus coeruleus; vlPAG: ventrolateral periaqueductal grey; PZ: parafacial zone; VLPO: ventrolateral preoptic area; PPT: pedunculopontine tegmentum; SLD: sublateral dorsal nucleus; vM: ventral medulla.

Numerous brain regions, cell types, and neural circuits involved in sleep–wake regulation have been identified to date (Figure 2, reviewed in [46]). The brainstem is an important brain region that contains many crucial neurons. Serotonergic neurons of the dorsal raphe nucleus (DRN), noradrenergic neurons and norepinephrinergic neurons of the locus coeruleus (LC), and dopaminergic (DA) neurons in the substantia nigra (SN) and ventral tegmental area (VTA) are wake-promoting neurons that innervate the thalamus and the entire forebrain. The ventral periaqueductal grey (vPAG) and parabrachial nucleus (PB) are also wake-active nuclei. Gamma-aminobutyric acid (GABA) producing neurons in the ventrolateral periaqueductal grey (vlPAG), parafacial zone (PZ), and glutamatergic neurons located ventromedial to the superior cerebellar peduncle (SCP) promote NREM sleep and suppress wakefulness and REM sleep. Cholinergic neurons in the pedunculopontine tegmentum (PPT) and laterodorsal tegmentum (LDT), and glutamatergic neurons in the sublateral dorsal nucleus (SLD) play an important role in promoting REM sleep generation and maintenance. The brainstem has been reported to be the first brain region in which hyperphosphorylated tau accumulates [28], which could be correlated with unstable wakefulness during daytime.

The ventrolateral preoptic area (VLPO)/intermediate nucleus, which is a sleep-active nucleus [47] that inhibits wake-promoting neurons [48,49,50] has shown reduced cell numbers in AD patients [51]. Cats with a lesion in the VLPO demonstrated fragmented sleep and reduced total sleep time [52], which is similar to the sleep disturbance that occurs in AD patients.

Degeneration in the basal forebrain (BF) of cholinergic nuclei is also observed in AD patients [53,54]. In the 1970s, a decrease in the presynaptic cholinergic marker choline acetyltransferase (ChAT) in the cerebral cortex of AD patients was reported [55,56], which was the first indication of a transmitter-based pathology in AD. Postmortem studies of AD patients demonstrated neuronal loss in cholinergic nuclei of the BF [57], which is the main source of acetylcholine in the cortex [58]. A magnetic resonance imaging (MRI) study demonstrated atrophy of cholinergic neurons in the BF of AD patients [53] and a correlation between its volume and amyloid burden level [54]. BF cholinergic neurons have been shown to be active during wakefulness and REM sleep [59,60]. Thus, neurodegeneration in the BF may underlie the reduction in REM sleep in AD patients.

Aβ and tau aggregation-mediated neurodegeneration also affects the orexin (hypocretin) system. Orexin is a wake-promoting neurotransmitter produced by the hypothalamus. It is crucial for stabilizing wakefulness and a deficit of orexin is the main pathology of narcolepsy and cataplexy [61]. Postmortem analysis has demonstrated that the number of hypothalamic orexin neurons is significantly decreased in AD patients [62]. The lack or reduction in orexin signaling appears to be associated with sleep problems in AD patients, particularly in those who experience excessive daytime sleepiness.

The amount of SWA during SWS has been shown to be decreased in AD patients compared with normal older individuals [31]. The precise mechanism of the disturbance in SWA during NREM sleep in AD patients is poorly understood. SWA is observed in EEG, and is accompanied by synchronization of the activities of a large number of cortical neurons oscillating between repetitive few hundred milliseconds of depolarized (active) and hyperpolarized (silent) periods [63]. The disruption of synaptic transmission caused by Aβ oligomers [20,21,22,23] and network dysfunction [64] may cause disturbances in oscillatory activities [43,65]. Animals show similar sleep alterations associated with aging as humans [66], and it has also been reported that SWA is disrupted in several AD models [67,68]. Some studies have suggested that the deficit of inhibitory tone may explain the disruption of SWA [69,70]. However, most studies have been conducted with animals under anesthesia, and little is known about SWA during naturally occurring sleep.

Although several brain regions and neuromodulatory system impairments could predict specific symptom of AD-associated sleep disorders, the precise neuronal mechanism corresponds to the sleep alteration are not fully elucidated. Further broad study with large scale data and experimental investigation using AD model animals is needed.

## 4. Sleep Alterations Preceding Clinical Stages of AD

Impairments in sleep are observed not only as a phenomenon accompanying AD, but even in the preclinical phase of AD, and has also been shown to be a risk factor of AD [71,72,73]. Cognitively normal older individuals who have highly fragmented sleep had a 1.5-fold increased risk of developing AD [74]. In addition, individuals who have the apolipoprotein E (ApoE) ε4 allele, which is a well-established genetic risk factor of AD [75,76], have been reported to have exacerbated sleep disruption compared with individuals without this allele [73,77].

Aβ begins to aggregate in the brain 10 to 15 years before the manifestation of clinical symptoms of AD [78]. It has been reported that Aβ levels correlate with sleep alterations in cognitively normal individuals [79,80]. Disruptions in NREM SWS begin in patients in the MCI stage [72]. SWS disturbance is therefore proposed to have potential as an early biomarker of AD [65]. Furthermore, Aβ deposits have been associated with decreased SWA during NREM sleep in cognitively normal older adults [81].

Tau deposition is observed even earlier than the aggregation of Aβ [28]. Tau tangles in cholinergic neurons significantly correlate with cognitive impairment, suggesting that disruption of the cortical cholinergic pathway is a very early event in the pathology of AD [82].

**Figure 3:**
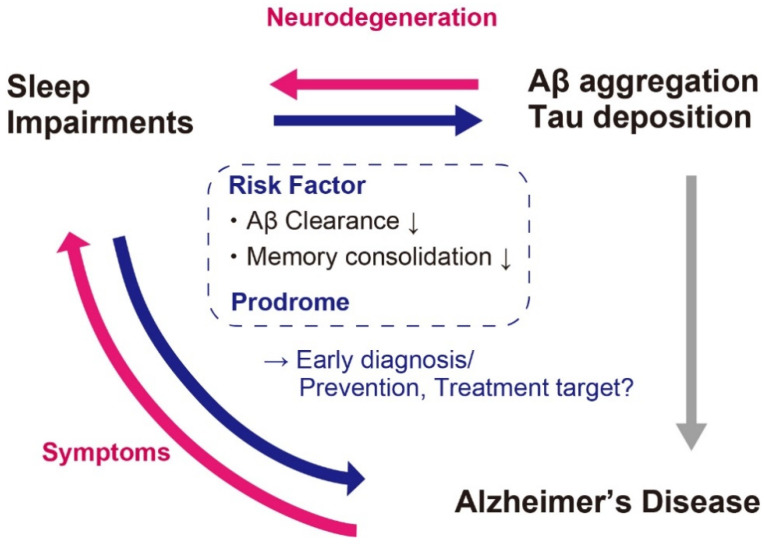
Association between sleep impairments and AD. Sleep impairments occurs not only as associated symptoms but also as prodrome. Moreover, sleep impairments can be a risk factor of Aβ aggregation and AD. It is therefore a promising candidate for the biomarker for early diagnosis and prevention or treatment target of AD.

It is therefore plausible that the Aβ aggregation and tau deposition-associated sleep impairments occur before the clinical stage or very early stage of AD (Figure 3).

## 5. Prevention and Treatments of AD Targeting Sleep Impairments

Sleep has been discussed as a possible target for AD prevention and treatments [43,45], with mainly two rationales; i.e., enhancing Aβ clearance during NREM sleep [83], and sleep-dependent memory consolidation and recovery of cognitive function [84,85]. The enhancement of NREM sleep or REM sleep, and the stabilization of sleep might be effective strategies for AD prevention and treatments (Figure 3).

Sleep deprivation has been reported to be associated with increased Aβ or tau level [86,87,88,89]. A study measuring Aβ levels in the cerebrospinal fluid (CSF) of individuals sampled through indwelling lumbar catheters demonstrated that the group subjected to sleep deprivation overnight showed increased Aβ_1-38_, Aβ_1-40_, and Aβ_1-42_ levels of 25% to 30% compared with the normal sleep group [87]. Furthermore, a study in healthy middle-aged men has reported that the CSF Aβ level was decreased by 6% after a night of unrestricted sleep, whereas this decrease was counteracted by a night of sleep deprivation [86]. CSF tau was also increased by more than 50% during sleep deprivation [88]. Aβ and tau have been shown to be increased during sleep deprivation also in mice [88].

It has been reported that SWS enhances Aβ clearance through the glymphatic system compared with the wake state [83]. Natural sleep is associated with a 60% increase in the interstitial space, resulting in an increase in the convective exchange of CSF [83]. Although this hypothesis is under debate [90], it may explain the sleep-dependent increase in Aβ and tau clearance.

NREM sleep is associated with decreased oxygen consumption, whereas the wake state is associated with increased oxygen and adenosine tri-phosphate (ATP) consumption, which results in higher metabolic stress [91,92]. Sleep deprivation significantly increases oxidative stress, which recovers upon sleep [93,94]. Oxidative stress promotes Aβ accumulation [95], and Aβ further promotes oxidative stress [96]. Insufficient sleep potentially induces difficulties in managing Aβ burden during the wake state, and exacerbates the Aβ accumulation associated with AD pathogenesis. Improvement of sleep would therefore provide a preventative effect of AD.

The most widely used therapeutic medications for AD are cholinesterase inhibitors, which enhance cholinergic transmission by inhibiting the enzyme acetylcholinesterase (AChE). Treatment of AD patients with the AChE inhibitor donepezil has been shown to increase the amount of REM sleep [97,98].

Aβ level is also associated with orexin [99]. The orexin receptor antagonist suvorexant was approved as a prescription drug for insomnia treatment in AD patients by the Food and Drug Administration (FDA) in the United States in 2020. In vivo microdialysis of mice has demonstrated that orexin antagonist infusion decreases Aβ level [100].

Other pharmacological treatments targeting the GABA system have been considered to be less promising as treatments for AD-associated sleep impairments. Benzodiazepine or non-selective GABA agonists such as zolpidem, which are commonly prescribed pharmacological treatments for sleep disorders, have been shown to disrupt memory in both humans and animals [101]. Moreover, the use of zolpidem has been reported to be associated with a higher risk of dementia [102].

Several nonpharmacological methods, including acoustic or mechanical approaches to improve sleep quality, have also been proposed. SWS has been shown to be enhanced by sound stimuli [103,104,105,106], and this acoustic stimulation indeed affects memory recall in some patients [104]. Rocking beds have also been shown to improve sleep by reducing sleep latency, increasing SWA, and reducing nocturnal arousal in both healthy adults and MCI patients [107,108,109]. They have also been reported to be associated with improved memory in healthy subjects [110].

Although more evidence needs to be provided, improvement of sleep seems a useful strategy for AD treatment.

## 6. PD and Abnormal Proteins

PD is the second most common neurodegenerative disease after AD. It is characterized by several movement features—such as rigidity, bradykinesia, and tremor. PD is pathologically linked to the formation of Lewy bodies and the loss of DA neurons predominantly in the substantia nigra pars compacta (SNpc), VTA, and vPAG in the midbrain [111,112].

Lewy bodies are mainly composed of aggregates of α-synuclein (α-Syn). α-Syn normally exists mainly in presynaptic terminals, and is involved in synaptic vesicle release [113]. Under pathological conditions, α-Syn undergoes post-translational modifications, including aberrant phosphorylation at serine 129, truncations, and oxidative damage, resulting in abnormal forms that facilitate its aggregation. There are several explanations for the specific vulnerability of SNpc DA neurons in PD, which are owing to their unique morphology and physiological properties [114]. SNpc DA neurons have long and highly branched unmyelinated axons [115], which may be associated with vulnerability to mitochondrial oxidative stress [116]. Furthermore, SNpc DA neurons show a slow and broad action potential, which maximizes calcium entry into the cell [117], and the increased calcium can promote the aggregation of α-Syn [118]. Possible effects of aggregated α-Syn on the MIT network, neuronal degeneration, trafficking defects, Golgi fragmentation, and neurotransmitter reuptake or release have been shown [119,120,121,122,123]. An in vitro study in cultured dorsal root ganglia (DRG) neurons reported that MIT-dependent trafficking is impaired by the overexpression of α-Syn [119]. Another study using adeno-associated virus (AAV) injection to overexpress human wild-type α-Syn into the SNpc in rats reported that dopamine reuptake starts to decrease 10 days after the injection, followed by reduced dopamine release starting at about 3 weeks after the AAV injection, with a delay in reaching the peak concentration [122]. Direct synaptic vesicle imaging in cells overexpressing α-Syn has demonstrated that the size of the synaptic vesicle recycling pool is reduced [123]. The pathological consequence appears to vary among wild-type α-Syn and different α-Syn mutants in the monomeric form [124,125]. Point mutations (A30P, E46K, H50Q, G51D, A53T, and A53E), and duplication or triplication of the α-Syn-encoding (SNCA) gene have been shown to cause familial forms of PD [126].

Although the pathogenesis of PD is becoming evident, it is yet difficult to prevent the onset of PD or eliminate the symptoms completely.

## 7. α-Syn and Sleep Impairments in PD

Sleep disorders are common nonmotor symptoms of PD patients, and it has been reported that 40% to 90% of PD patients are affected by sleep disorders [127,128]. Decreased sleep efficiency, and REM behavior disorder (RBD), which is defined as REM sleep without atonia and excessive daytime sleepiness or narcolepsy-like symptoms, are particularly noticeable in PD patients [129,130]. PD patients also often experience insomnia, nightmares, nocturia, restless leg syndrome, and obstructive sleep apnea [129,131,132].

Similar to the sleep impairments experienced by AD patients, neurodegeneration in the brain regions involved in sleep–wake regulation results in various sleep disorders in PD patients. Neuropathology in the early stage of PD is considered to occur in the dorsal motor nucleus of the glossopharyngeal and vagal nerves, and in the anterior olfactory nucleus in the olfactory bulb, followed by that in the raphe nuclei, gigantocellular reticular nucleus (GiV), and LC, and then further expands to the SNpc, thalamus, and neocortex in an ascending pathway [111,133]. RBD, for example, is associated with dysfunction of the SLD and magnocellular reticular formation (MCRF) structures. In the healthy condition, REM atonia occurs by inhibitory input to spinal motor neurons from the SLD [134]. SLD glutamatergic neurons project to the ventral medulla (vM), including the GiV, which in turn inhibits spinal motor neurons and induces muscle atonia (Figure 2). RBD is caused by the lack of SLD excitatory input to the vM during REM sleep [134].

The loss of orexin cells has been reported to increase with disease progression in PD [135]. In addition, CSF orexin level is decreased in PD patients [136]. Indeed, narcolepsy-like symptoms have been reported in some patients with PD [137].

Circadian rhythm consists of a network of central and peripheral daily oscillations—including body temperature, endocrine function, and blood pressure—through autonomous rhythms, and is another important factor in the sleep–wake cycle [138]. The central clock of the circadian rhythm is located in the suprachiasmatic nucleus (SCN), which receives light information form the retina, and is known to be damaged in PD patients as a result of dopaminergic retinal degeneration [139]. SCN neurons mainly project to the hypothalamic region, which modulates the endocrine system and other circadian oscillation networks. Melatonin output is regulated by SCN output, and reaches a peak in the evening and promotes sleep. Melatonin is produced by the pineal gland. PD patients have disrupted circadian rhythms of melatonin secretion, and the lower amplitude of the melatonin rhythms correlates with excessive daytime sleepiness [140].

Sleep impairment associated with PD is an important component of PD pathology that causes severe impact on the life of the patients.

## 8. Sleep Impairments Preceding Clinical PD

Sleep disturbance has been reported to occur preceding the clinical onset of movement disorder in some studies [141,142]. Monitoring of the activity of healthy adults using actigraphy demonstrated that greater sleep fragmentation is associated with the presence of Lewy body pathology, loss of SN neurons, and higher odds of pathological diagnosis of PD [141]. It has also been reported that 73.5% of people who had RBD converted to an overt neurodegenerative syndrome during a 12-year follow-up period [142]. Another study showed that lower sleep quality and shorter sleep duration were associated with a higher risk of parkinsonism [143]. Excessive daytime sleepiness also seems to predict PD. A large population-based study has reported that those who had daytime napping of longer than 1 h per day that suggest excessive daytime sleepiness had an increased risk of developing PD [144].

Sleep impairments preceding the motor disorders of PD have also been observed in PD model mice. 1-Methyl-4-phenyl-1,2,3,6-tetrahydropyridine (MPTP) is a neurotoxin against DA neurons, which is used to create animal models of PD. Mice show robust disruption of their sleep–wake architecture after MPTP administration, with dysregulation of REM sleep and increased daytime sleepiness occurring before motor symptoms [145]. Furthermore, transgenic mice expressing SNCA genes with the A53T mutation showed RBD at 5 months of age in the absence of any motor symptoms [146]. Another PD mouse model created by injecting preformed α-Syn fibrils into the SLD showed RBD-like behavior followed by α-synucleinopathy and neurodegeneration [147].

**Figure 4:**
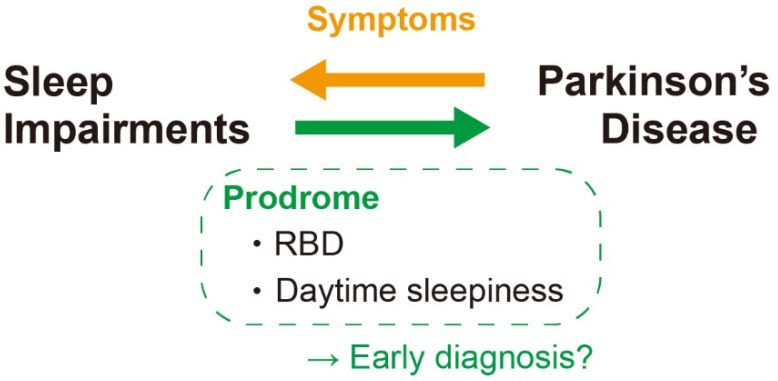
Association between sleep impairments and PD. RBD and daytime sleepiness are two principal sleep impairments in PD patient that is reported to be happened even as a prodrome. It could be useful as a biomarker for the early diagnosis of PD. RBD: REM behavior disorder.

Therefore, sleep alterations—particularly the excessive daytime sleepiness and RBD—might be a prodromal feature of PD (Figure 4).

## 9. Sleep Disorders as a Prevention and Treatment Target of PD

One of the reasons for the difficulty of PD treatment is the late diagnosis since the characteristic motor symptoms appear only after considerable progression of PD [148]. Early diagnosis is therefore an urgent matter to start the treatment at the early phase of the disease and prevent or delay the onset of severe motor symptoms. The sleep impairments at the early phase of PD must be useful for that (Figure 4).

Pharmacological therapies for PD patients target the dopamine system, which is the main site of dysfunction in parkinsonism. The dopamine precursor L-dopa, dopamine agonists, or monoamine oxidase (MAO)-B inhibitors, which inhibit dopamine degradation, are commonly used. L-dopa therapy has been suggested to improve retinal dopamine content in PD patients [139].

Melatonin supplementation has been reported to ameliorate RBD [149]. PD patients experiencing sleep disturbance reported significant improvement in total night time sleep upon melatonin treatment, although the authors concluded that the effect was modest [150]. Another study reported that melatonin improves subjective sleep quality, although no changes were observed in sleep abnormalities on polysomnography (PSG), or in motor dysfunction [151].

Orexin is another promising target for the treatment of PD. A study in PD model mice induced by MPTP injection showed improvements in motor activity and spatial memory by orexin-A administration [152]. In PD model rats created by intracerebroventricular injection of 6-hydroxydopamine (6-OHDA), another widely-used neurotoxin against DA neurons, it was reported that orexin-A administration improved motor function [153].

## 10. Conclusions

Sleep disturbance is a common symptom associated with AD and PD, and has a considerable impact on the quality of life of patients and their caregivers. Sleep dysfunction appears to have a reciprocal relationship with neurodegeneration, which has recently gained attention, and may be a potential target for effective therapies or a biomarker for the early diagnosis of these diseases. Moreover, both further large-scale studies and elucidation of the precise mechanism involved may be required to clarify the contribution of insufficient sleep to the development of neurodegenerative disorders.

## Data Availability

No new data were created or analyzed in this study. Data sharing is not applicable to this article.
